# Longitudinal telomere length shortening and cognitive and physical decline in later life: The Lothian Birth Cohorts 1936 and 1921

**DOI:** 10.1016/j.mad.2016.02.004

**Published:** 2016-03

**Authors:** Sarah E. Harris, Riccardo E. Marioni, Carmen Martin-Ruiz, Alison Pattie, Alan J. Gow, Simon R. Cox, Janie Corley, Thomas von Zglinicki, John M. Starr, Ian J. Deary

**Affiliations:** a Centre for Cognitive Ageing and Cognitive Epidemiology, University of Edinburgh, 7 George Square, Edinburgh EH8 9JZ, UK; b Medical Genetics Section, University of Edinburgh Centre for Genomic and Experimental Medicine and MRC Institute of Genetics and Molecular Medicine, Western General Hospital, Crewe Road, Edinburgh EH4 2XU, UK; c Queensland Brain Institute, The University of Queensland, Brisbane 4072, QLD, Australia; d Institute for Ageing, Newcastle University, Campus for Ageing and Vitality, Newcastle upon Tyne NE4 5PL, UK; e Department of Psychology, University of Edinburgh, 7 George Square, Edinburgh EH8 9JZ, UK; f Department of Psychology, School of Life Sciences, Heriot-Watt University, Edinburgh EH14 4AS, UK; g Alzheimer Scotland Dementia Research Centre, University of Edinburgh, 7 George Square, Edinburgh EH8 9JZ, UK

**Keywords:** Telomeres, Cognition, Cognitive ageing, Physical ageing

## Abstract

Telomere length is hypothesised to be a biological marker of both cognitive and physical ageing. Here we measure telomere length, and cognitive and physical abilities at mean ages 70, 73 and 76 years in the Lothian Birth Cohort 1936 (LBC1936), and at mean ages 79, 87, 90 and 92 years in the Lothian Birth Cohort 1921 (LBC1921). We investigate whether telomere length change predicts change in cognitive and physical abilities. In LBC1936 telomere length decreased by an average of 65 base pairs per year and in LBC1921 by 69 base pairs per year. However, change in telomere length did not predict change in cognitive or physical abilities. This study shows that, although cognitive ability, walking speed, lung function and grip strength all decline with age, they do so independently of telomere length shortening.

## Highlights

• Telomere length is hypothesised to be a biological marker of ageing.
• In LBC1936 and LBC1921 telomere length declines with age.
• Telomere length change did not predict change in cognitive and physical abilities.

## 1 Introduction

Determining the biological factors that influence both cognitive and physical decline in later life is an important challenge facing researchers today (Blazer et al., 2015, den Ouden et al., 2011). Telomeres are nucleo-protein complexes at the end of eukaryotic chromosomes. They protect the ends of chromosomes, but shorten each time a somatic cell replicates (Harley et al., 1990, Lindsey et al., 1991). Environmental factors also contribute to accelerated decline in telomere length. These include low socio-economic status, smoking, oxidative stress, and psychological stress (Valdes et al., 2005, von Zglinicki, 2002, Robertson et al., 2013). Telomere length decreases with age and a systematic review determined that the correlation between telomere length and chronological age is about −0.3 (Muezzinler et al., 2013). Leukocyte telomere length has previously been associated with a number of traits and diseases in older age including cognitive abilities (Harris et al., 2006, Harris et al., 2012, Yaffe et al., 2009, Der et al., 2012, Ma et al., 2013), dementia (Grodstein et al., 2008, Martin-Ruiz et al., 2006, Panossian et al., 2003, von Zglinicki et al., 2000), physical health (Gardner et al., 2013, Woo et al., 2014, Masi et al., 2014, Baylis et al., 2014) and obesity (Valdes et al., 2005, Njajou et al., 2012), and has been hypothesised to be a biological marker of ageing (von Zglinicki and Martin-Ruiz, 2005). However, a systematic review concluded that current results were equivocal and that more studies, including longitudinal studies, were required that assessed telomere length and ageing-related functional measures (Mather et al., 2011). Longitudinal studies have the potential to measure age-related decline in telomere length, and cognitive and physical abilities more accurately than cross-sectional studies and also allow the investigation of the change of multiple variables in parallel with each other.

There are many studies that show lower childhood cognitive ability is associated with poorer health and more illness in adulthood and older age, and to earlier mortality from all causes and from several specific causes, such as cardiovascular disease (Deary et al., 2010). Early life IQ has previously been associated with telomere length in midlife (Schaefer et al., 2015). The mechanism of the childhood cognition-illness/death association is not understood, but it is possible that telomeres might provide a biomarker of how lifestyle has affected the body.

We previously reported mostly-null cross-sectional associations between telomere length and cognitive function, walking speed, lung function, and grip strength in the Lothian Birth Cohorts of 1921 and 1936 (LBC1921 and LBC1936) (Harris et al., 2006, Harris et al., 2012). More recently, we showed that the same cognitive and physical abilities decline on average between ages 70 and 76 years in LBC1936 (Marioni et al., 2015). Here, we report longitudinal analyses investigating whether decline in telomere length predicts cognitive and physical decline in the Lothian Birth Cohorts. We also investigate whether baseline telomere length influences subsequent decline in cognitive and physical abilities. Finally, we test whether cognitive ability measured in childhood is related to telomere length decline in later life.

## 2 Materials and methods

### 2.1 Lothian Birth Cohort 1936 (LBC1936)

LBC1936 consists of 1091 (548 men and 543 women) surviving members of the Scottish Mental Survey of 1947 (Scottish Council for Research in Education, 1949). At approximately age 11 years most took a valid mental ability test, the Moray House Test version 12 (MHT). At a mean age of 69.5 years (SD 0.8) they were recruited to a study to determine influences on cognitive ageing (Deary et al., 2007, Deary et al., 2012a). They underwent a series of cognitive and physical tests. Two further waves of cognitive and physical tests have occurred at mean ages 73 and 76 years. DNA was extracted from peripheral blood leukocytes at ages 70, 73 and 76 years from which telomere length was measured. Cognitive tests taken at each of the three waves included six Wechsler Adult Intelligence Scale-IIIUK (WAIS-III) (Wechsler, 1998) non-verbal subtests (matrix reasoning, letter number sequencing, block design, symbol search, digit symbol, and digit span backward). From these six cognitive tests a general fluid cognitive factor (*g*_f_) was derived. The scores from the first unrotated component of a principal components analysis were extracted and labelled as *g*_f_. This component explained 52% of the variance, with individual test loadings ranging between 0.65 and 0.72. Physical trait measures included time taken to walk six metres at normal pace, grip strength measured with a Jamar Hydraulic Hand Dynamometer (all subjects had three trials with the dominant hand; the best of the three trials was used), and forced expiratory volume from the lungs in one second (FEV_1_) measured using a microspirometer (the best of the three trials was used).

### 2.2 Lothian Birth Cohort 1921 (LBC1921)

LBC1921 consists of 550 (234 men and 316 women) surviving members of the Scottish Mental Survey of 1932 (Scottish Council for Research in Education, 1933). At approximately age 11 years most took a valid mental ability test, the MHT. At a mean age of 79.1 years (SD 0.6) they were recruited to a study to determine influences on cognitive ageing (Deary et al., 2004, Deary et al., 2012b). They underwent a series of cognitive and physical tests. Four further waves of cognitive and physical tests have occurred at mean ages 83, 87, 90 and 92 years. DNA was extracted from peripheral blood leukocytes at ages 79, 87, 90 and 92 years from which telomere length was measured. Cognitive tests taken at each of these four waves included Raven’s Progressive Matrices (Raven et al., 1977), Verbal Fluency (Lezak, 1995) and Logical Memory (Wechsler, 1987). From these three cognitive tests a general fluid cognitive factor (*g*_f_) was derived using principal component analysis. The scores from the first unrotated component were extracted and labelled as *g*_f_. This component explained 53% of the variance, with individual test loadings ranging between 0.65 and 0.73. Physical trait measures included time taken to walk six metres at normal pace, grip strength measured with a Jamar Hydraulic Hand Dynamometer (all subjects had three trials with the dominant hand; the best of the three trials was used) and forced expiratory volume from the lungs in one second (FEV_1_) measured using a microspirometer (the best of the three trials was used).

Ethics permission for the LBC1936 was obtained from the Multi-Centre Research Ethics Committee for Scotland (Wave 1: MREC/01/0/56), the Lothian Research Ethics Committee (Wave 1: LREC/2003/2/29), and the Scotland A Research Ethics Committee (Waves 2 and 3: 07/MRE00/58). Ethics permission for the LBC1921 was obtained from the Lothian Research Ethics Committee (Wave 1: LREC/1998/4/183; Wave 3:1702/98/4/183) and the Scotland A Research Ethics Committee (Waves 4 and 5:10/S1103/6). All persons gave their informed consent prior to their inclusion in the study.

### 2.3 Telomere length measurement

DNA was extracted from whole blood by standard procedures at the Wellcome Trust Clinical Research Facility Genetics Core at the Western General Hospital, Edinburgh. Telomere length was measured using a quantitative real-time polymerase chain reaction (PCR) assay (Martin-Ruiz et al., 2004). The intra-assay coefficient of variation was 2.7% and the inter-assay coefficient of variation was 5.1%. Four internal control DNA samples were run within each plate to generate absolute telomere lengths and to correct for plate to plate variation. These internal controls are cell lines of known absolute telomere length, 6.9 kb, 4.03 kb, 2.0 kb and 1.32 kb respectively, whose relative ratio values (telomere starting quantity/glyceraldehyde 3-phosphate dehydrogenase starting quantity) were used to generate a regression line by which values of relative telomere length for the actual samples were converted into absolute telomere lengths. The correlation between relative telomere length and absolute telomere length was 0.8. Measurements were performed in quadruplicate and the mean of the measurements used. PCRs were performed on an Applied Biosystems (Pleasonton, CA, USA) 7900HT Fast Real Time PCR machine.

### 2.4 Statistical analyses

Linear mixed models were used to determine if telomere length and cognitive and physical abilities changed over time. One individual with chronic lymphocytic leukaemia was removed from the LBC1936 analyses. Covariates included age (centred at the minimum value) as the time scale, sex, for telomere length white blood cell counts (lymphocyte, basophil, neutrophil, eosinophil and monocyte) and for physical abilities height. Individual participant number was included as a random effect. Baseline telomere length was added as a fixed effect interaction with age to test if it predicted decline in cognitive and physical abilities. Age 11 MHT score (corrected for age at time of testing and sex) was then added as a fixed effect interaction with age to test if it predicted decline in telomere length. In LBC1921, linear regression was used to determine if age 11 MHT score was associated with telomere length at age 79 years.

Linear mixed models were then used to investigate if telomere length change predicted change in cognitive and physical abilities. Again covariates included age (centred at the minimum value) as the time scale, sex, white cell counts and for physical abilities height. Individual participant number was included as a random effect. Linear mixed models were performed in R using the lme4 and lmerTest packages (Kuznetsova et al., 2013, Bates et al., 2013).

## 3 Results

Descriptive statistics for telomere length, general fluid cognitive ability (*g*_f_), time taken to walk six metres, forced expiratory volume in one second (FEV_1_) and grip strength for LBC1936 waves 1 (age ∼70 years), 2 (age ∼73 years) and 3 (age ∼79 years) are shown in Table 1, and for LBC1921 waves 1 (age ∼79 years), 3 (age ∼87 years), 4 (age ∼90 years) and 5 (age ∼92 years) are shown in Table 2.

In LBC1936, mean telomere length decreased with age. In LBC1921, mean telomere length remained relatively stable between ages 79 and 87 years and then decreased with age. In both cohorts *g*_f_, FEV_1_ and grip strength all decreased with age and time taken to walk six metres increased. Mean age, telomere length and FEV_1_ did not differ between all individuals who participated in a particular wave of testing and those who returned for later waves of testing. Individuals who returned for further waves of testing generally had a slightly higher *g*_f_, a faster walk time and a stronger grip strength on the first occasion of testing.

**Fig. 1:**
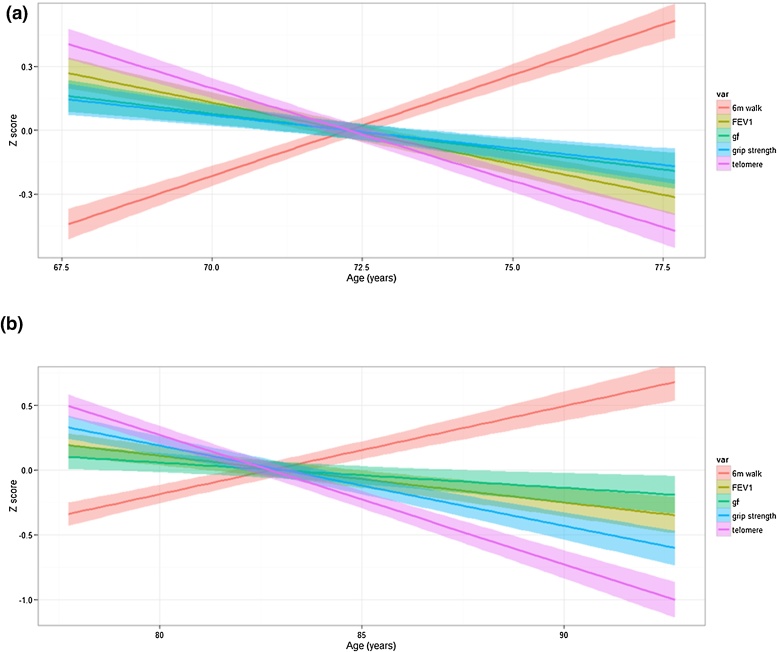
Mean trajectory plots for change in telomere length, general cognitive ability (*g*_f_) six metre walk time, forced expiratory volume in 1 s (FEV_1_), and grip strength for (a) LBC1936 and (b) LBC1921.

Mean trajectory plots for change in telomere length, *g*_f_, six metre walk time, FEV_1_, and grip strength for LBC1936 and LBC1921 are shown in Fig. 1.

In LBC1936, a linear mixed model indicated that telomere length decreased by 64.8 base pairs (bp) per year (*p* < 2 × 10^−16^), which is 1.5% of the mean telomere length at age 70 years. Telomeres were 177.9 bp longer in males than females (*p* = 4.66 × 10^−7^). Telomere length decreased with increasing lymphocyte cell count (*p* = 9.5 × 10^−4^), but was not associated with any other white blood cell count. As previously shown^27^, *g*_f_ decreased by 0.05 standard deviations per year, 6 m walk time increased by 0.15 s per year, FEV_1_ decreased by 0.05 L per year, and grip strength decreased by 0.04 kg per year (all *p* values < 2 × 10^−16^). There was no evidence to suggest that baseline telomere length was associated with trajectory of decline in cognitive and physical abilities (all *p*-values > 0.2). Age 11 Moray House Test (MHT) score was not linked to differences in change in telomere length (*p* = 0.88).

In LBC1921, a linear mixed model indicated that telomere length decreased by 69.3 bp per year (*p* < 2 × 10^−16^), which is 1.7% of the mean telomere length at age 79 years. Telomeres were 256.9 bp longer in males than females (*p* = 1.2 × 10^−8^). Telomere length was not associated with white blood cell counts (all *p* values > 0.05). *g*_f_ decreased by 0.05 standard deviations per year, 6 m walk time increased by 0.27 s per year, FEV_1_ decreased by 0.03 L per year, and grip strength decreased by 0.74 kg per year (all *p* values < 2 × 10^−16^). Baseline telomere length was not associated with decline in cognitive or physical abilities (all *p*-values > 0.1). Age 11 Moray House Test (MHT) score was linked to the amount of telomere length change such that, for a standard deviation increase in age 11 cognitive ability score, there was a 9.7 bp greater decrease in telomere length per year (*p* = 0.044). Age 11 MHT score was not associated with telomere length at age 79 years (*p* = 0.79).

In LBC1936 and LBC1921 there was no evidence to suggest that differences in telomere length change correlated with differences in change in cognitive or physical abilities (all *p*-values > 0.1) (Table 3).

## 4 Discussion

This study indicates that, in both LBC1936 and LBC1921, mean telomere length decreased by ∼65 bp per year, which is just under 2% of the mean telomere length at baseline. This is slightly higher than that reported for other longitudinal studies, which ranged from 32 to 46 bp per year (Muezzinler et al., 2013). Cognitive and physical abilities also decreased during this period. Telomere length at baseline was not associated with decline in cognitive or physical abilities between the ages of 70 and 76 (LBC1936), or 79 and 92 (LBC1921) years. In LBC1921 childhood cognitive ability was linked to the amount of telomere length change such that, individuals with a higher childhood cognitive ability underwent a greater decrease in telomere length per year in later life. The rate of decrease in telomere length did not correlate with the rate of decrease in cognitive and physical abilities in either cohort.

As far as we aware this is the first longitudinal study, measuring at least three time points, to investigate if telomere length decline is associated with cognitive and physical decline. A recent meta-analysis based on two time points also found little evidence for telomere length decline as biomarker for physical decline (Gardner et al., 2013). Our results largely agree with previously published cross-sectional findings that telomere length does not associate with cognitive and physical ability (Yaffe et al., 2009, Harris et al., 2012, Bendix et al., 2011). The results confirm the conclusions from a number of previous papers that telomere length is not informative as a biomarker for multiple dimensions of age-related risks including cognitive decline, multi-morbidity and mortality (Cawthon et al., 2003, Martin-Ruiz et al., 2005, Martin-Ruiz et al., 2011). In LBC1936 and LBC1921, telomere length was longer in males than in females, which contradicts many previous studies (Gardner et al., 2014). However, this may reflect the fact that life-expectancy of women is higher than men. Due to the older-age range of the Lothian Birth Cohorts, the men are typically much healthier than those of a similar age in the general population, whereas the women may be more representative of women of a similar age in the general population (Harris et al., 2012). Also a meta-analysis study looking at different methods of measuring telomere length concluded that only the Southern blot method generates results where women have longer telomeres than men (Gardner et al., 2014). Interestingly, mean telomere length at age 79 years in LBC1921 (4.1 kb) was longer than mean telomere age at age 76 years in LBC1936 (3.7 kb). This may be due to the selection of relatively healthier participants into a study at age 79 years (Deary et al., 2012a) compared to those aged 76 years who were already involved in a study. However, the physical ability data does not support this theory e.g., mean grip strength at age 79 years in LBC1921 (26.5 kg) was less than mean grip strength in LBC1936 at age 76 years (27.9 kg). Also, a recent study showed that although there is a negative correlation between age and telomere length up to age 75 years, after 75 years the correlation becomes positive (Lapham et al., 2015).

In LBC1936 higher lymphocyte count was associated with shorter telomeres, indicating that white blood cell distribution may be a predictor of telomere length, as shown previously (Glei et al., 2015). In LBC1921, age 11 cognitive ability was linked to telomere length change such that individuals with a higher Moray House Test score at age 11 years showed a greater decline in telomere length in later life. This was not due to individuals with higher age 11 cognitive ability scores having longer telomeres at age 79 years. Age 11 cognitive ability scores did not influence telomere length change in LBC1936 and the significant result in LBC1921 may be due to type 1 error. Therefore, this finding needs replicating in another study before being considered further.

Strengths of this study include the longitudinal nature, with measurements at three and four time points of the telomeres and the cognitive and physical abilities in two narrow-age cohorts whose combined age periods range from 70 to 92 years. A further strength is that our absolute values of telomere length were generated using four internal controls which are cell lines of known absolute telomere length, whose relative ratio values were used to generate a regression line by which values of relative telomere length for the actual samples were converted into absolute telomere lengths. This allowed us to accurately correct for plate to plate variations as it is well known that the quantitative real-time PCR assay method is sensitive to efficiency variations between very long or very short telomere amplifications. PCR efficiency is not the same for samples with long telomeres compared to samples with short telomeres. A disadvantage of the study is the relatively short time period between each wave of testing. As with all longitudinal studies, there was attrition, though the statistical method used all the available data. Selection bias due to differential mortality is a common limitation in longitudinal studies. However, in this study baseline telomere length and FEV_1_ did not differ between individuals who did and did not return for later waves of testing. Individuals who returned for further waves of testing generally had a slightly higher *g*_f_, a faster walk time and a stronger grip strength on the first occasion of testing, indicating some selection bias. This may reduce the power of the study to detect associations between telomere length shortening and cognitive and physical decline. A further limitation of the study is that the sample sizes of the cohorts, particularly at later waves, is perhaps not large enough to detect a correlation between telomere length shortening and decline in cognitive and physical abilities. The relative health of the cohorts also reduces the variance of the cognitive and physical phenotypes relative to the general population.

## 5 Conclusion

We find that, although telomere length, and cognitive and physical abilities all show mean decline with age in LBC1936 from age 70 to 76, and in LBC1921 from age 79 to 92, the shortening of telomeres is independent from the observed decline in cognitive and physical abilities.

## Figures and Tables

**Table 1:** Summary descriptive data for LBC1936. *g*_f_ = general cognitive ability, FEV_1_ = forced expiratory volume in one second.

	Age 70						Age 73				Age 76	
	All		Age 73 completers		Age 76 completers		All		Age 76 completers		All	
	N	Mean (SD, range)	N	Mean (SD, range)	N	Mean (SD, range)	N	Mean (SD, range)	N	Mean (SD, range)	N	Mean (SD, range)
Age (years)	1091	69.5 (0.8, 67.6–71.3)	866	69.5 (0.8, 67.6–71.3)	697	69.5 (0.8, 67.6–71.3)	866	72.5 (0.7, 70.9–74.2)	697	72.5 (0.7, 70.9–74.2)	697	76.2 (0.7, 74.6–77.7)
Telomere length (kb)	1070	4.2 (0.6, 2.7–7.1)	855	4.2 (0.6, 2.7–7.1)	691	4.2 (0.6, 2.7–7.1)	844	4.0 (0.7, 1.9–9.4)	678	4.0 (0.7, 1.9–9.4)	689	3.7 (0.7, 1.9–7.2)
*g*_f_	1072	0.04 (1.0, −3.5–3.0)	853	0.12 (1.0, −3.5–3.0)	687	0.19 (1.0, −3.5–3.0)	856	0.02 (0.98, −3.4–3.1)	690	0.11 (0.96, −2.7–3.1)	668	−0.08 (1.0, −3.0–3.1)
6 m walk time (s)	1085	3.9 (1.2, 1.1–14.7)	863	3.8 (1.1, 1.1–14.7)	695	3.7 (1.0, 1.1–14.7)	860	4.4 (1.3, 1.6–16.0)	692	4.2 (1.1, 1.6–16.0)	692	4.7 (0.5, 1.5–15.3)
FEV_1_ (L)	1085	2.4 (0.7, 0.5–5.1)	863	2.4 (0.7, 0.7–5.1)	695	2.4 (0.7, 0.7–5.1)	856	2.3 (0.7, 0.4–5.2)	692	2.3 (0.7, 0.4–5.2)	690	2.1 (0.6, 0.6–4.1)
Grip strength (Kg)	1086	29.6 (10.2, 6.0–60.0)	864	30.1 (10.0, 6.0–60.0)	696	30.4 (10.1, 6.0–60.0)	865	29.1 (9.5, 2.0–59.0)	696	29.4 (9.5, 2.0–59.0)	691	27.9 (9.6, 1.0–55.0)

**Table 2:** Summary descriptive data for LBC1921. *g*_f_ = general cognitive ability, FEV_1_ = forced expiratory volume in one second.

	Age 79								Age 87						Age 90				Age 92	
	All		Age 87 completers		Age 90 completers		Age 92 completers		All		Age 90 completers		Age 92 completers		All		Age 92 completers		All	
	N	Mean (SD, range)	N	Mean (SD, range)	N	Mean (SD, range)	N	Mean (SD, range)	N	Mean (SD, range)	N	Mean (SD, range)	N	Mean (SD, range)	N	Mean (SD, range)	N	Mean (SD, range)	N	Mean (SD, range)
Age (yrs)	550	79.1 (0.6, 77.7–80.6)	233	79.1 (0.6, 77.8–80.6)	128	79.1 (0.6, 77.8–80.6)	59	79.1 (0.6, 77.9–80.1)	235	86.6 (0.4, 85.7–87.5)	129	86.6 (0.4, 85.7–87.5)	59	86.6 (0.4, 85.7–87.4)	129	90.1 (0.1, 89.2–90.8)	59	90.1 (0.1, 89.9–90.4)	59	92.1 (0.3, 91.3–92.7)
Tel length (kb)	497	4.1 (0.4, 1.9–5.6)	186	4.1 (0.4, 1.9–5.6)	99	4.2 (0.4, 3.3–5.6)	45	4.1 (0.4, 3.3–5.3)	146	4.2 (0.6, 1.9–5.8)	91	4.2 (0.5, 1.9–5.3)	45	4.1 (0.6, 1.9–5.3)	92	3.2 (0.7, 1.4–5.3)	57	3.1 (0.6, 1.7–4.6)	58	2.9 (0.5, 1.0–4.2)
*g*_f_	538	0.07 (0.9, −2.6–2.9)	231	0.34 (0.9, −1.8–2.9)	128	0.45 (0.9, −1.7–2.9)	59	0.43 (0.9, −1.7–2.4)	202	-0.08 (1.1, −3.2–3.3)	126	0.21 (0.9, −2.3–2.1)	59	0.33 (0.8, −1.5–1.9)	118	-0.15 (1.1, −2.3–2.3)	57	0.04 (1.0, −2.0–2.2)	54	-0.12 (1.0, −1.9–2.4)
6 m walk time (s)	541	4.7 (1.9, 1.8–27)	232	4.4 (1.4, 2.3–12.1)	128	4.3 (1.3, 2.5–9.9)	59	4.3 (1.3, 2.5–9.9)	192	6.7 (5.1, 2.8–56.1)	121	5.9 (2.9, 2.8–22.5)	59	5.7 (2.8, 2.9–22.5)	97	7.2 (4.8, 3.4–46.4)	58	6.5 (2.9, 3.4–19.1)	57	7.7 (2.7, 3.6–13.9)
FEV_1_ (L)	544	1.9 (0.6, 0.5–3.9)	233	2.0 (0.6, 0.6–3.7)	128	2.0 (0.6, 0.7–3.3)	59	2.1 (0.5, 0.9–3.1)	206	1.8 (0.6, 0.5–3.9)	129	1.8 (0.6, 0.5–3.9)	59	1.8 (0.5, 0.9–2.9)	128	1.7 (0.5, 0.5–3.2)	59	1.7 (0.5, 0.7–2.9)	59	1.5 (0.5, 0.6–2.6)
Grip strength (Kg)	544	26.5 (9.1, 4.0–59.0)	233	28.0 (9.6, 11.0–59.0)	128	28.7 (9.3, 14.0–53.0)	59	28.5 (9.6, 15.0–51.0)	205	21.5 (8.8, 0.0–44.0)	127	22.3 (8.9, 1.0–44.0)	57	23.0 (9.1, 6.0–44.0)	128	21.0 (8.3, 6.0–46.0)	59	21.6 (7.8, 6.0–45.0)	59	18.7 (8.9, 1.0–42.0)

**Table 3:** Effect of change in telomere length on change in cognitive and physical abilities. *g*_f_ = general cognitive ability, FEV_1_ = forced expiratory volume in one second.

	LBC1936			LBC1921		
	Beta	95% CI	*P*	Beta	95% CI	*P*
*g*_f_	−1.7 × 10^−3^	−8 × 10^−3^–0.01	0.74	0.0011	−0.02–0.02	0.91
6 m walk time (s)	6.2 × 10^−3^	−0.02–0.03	0.59	0.010	−0.06–0.08	0.78
FEV_1_ (L)	2.0 × 10^−3^	−4 × 10^−3^–8 × 10^−3^	0.50	0.0029	−4 × 10^−3^–0.01	0.43
Grip strength (Kg)	−0.03	−0.1–0.07	0.47	−0.035	−0.1–0.07	0.53
